# Oxidized Cell-Free DNA Role in the Antioxidant Defense Mechanisms under Stress

**DOI:** 10.1155/2019/1245749

**Published:** 2019-07-08

**Authors:** A. D. Filev, G. V. Shmarina, E. S. Ershova, N. N. Veiko, A. V. Martynov, M. A. Borzikova, A. A. Poletkina, O. A. Dolgikh, V. P. Veiko, A. A. Bekker, A. V. Chirkov, Z. N. Volynshchikov, A. S. Deviataikina, D. M. Shashin, V. K. Puretskiy, V. J. Tabakov, V. L. Izhevskaya, S. I. Kutsev, S. V. Kostyuk, P. E. Umriukhin

**Affiliations:** ^1^Research Centre for Medical Genetics (RCMG), Moskvorechie 1, Moscow 115478, Russia; ^2^I.M. Sechenov First Moscow State Medical University (Sechenov University), Mohovaya str., 11 str.5, 125007 Moscow, Russia; ^3^G.N. Gabrichevsky Institute of Epidemiology and Microbiology, Admiral Makarov str. 10, 125367 Moscow, Russia; ^4^A.N. Bach Institute of Biochemistry, bld 33-2, Leninsly Prospect, Moscow 119071, Russia; ^5^Pirogov Russian National Research Medical University (RNRMU), Ostrovitianov str. 1, Moscow 117997, Russia; ^6^P.K. Anokhin Institute of Normal Physiology, Baltiyskaya str. 8, 125315 Moscow, Russia

## Abstract

The present study focuses on the investigation of the oxidized cell-free DNA (cfDNA) properties in several experimental models, including cultured cerebellum cells, peripheral blood lymphocytes (PBL), plasma, and hippocampus under an acute and chronic unpredictable stress model in rats. Firstly, our study shows that Spectrum Green fluorescence-labeled oxidized cfDNA fragments were transferred into the cytoplasm of 80% of the cerebellum culture cells; meanwhile, the nonoxidized cfDNA fragments do not pass into the cells. Oxidized cfDNA stimulates the antioxidant mechanisms and induction of transcription factor NRF2 expression, followed by an activation of NRF2 signaling pathway genes—rise of *Nrf2* and *Hmox1* gene expression and consequently NRF2 protein synthesis. Secondly, we showed that stress increases plasma cfDNA concentration in rats corresponding with the duration of the stress exposure. At the same time, our study did not reveal any significant changes of 8-oxo-7,8-dihydro-2′-deoxyguanosine (8-oxodG) level in PBL of rats under acute or chronic stress, probably due to the significantly increased *Nrf2* expression, that we found in such conditions. 8-oxodG is one of the most reliable markers of DNA oxidation. We also found an increased level of 8-oxodG in the hippocampal homogenates and hippocampal dentate gyrus in rats subjected to acute and chronic stress. Taken together, our data shows that oxidized cfDNA may play a significant role in systemic and neuronal physiological mechanisms of stress and adaptation.

## 1. Introduction

DNA that circulates in the blood plasma (serum) is called cell-free DNA (cfDNA) or extracellular (ecDNA) [1–3]. The most widely accepted hypothesis is that the main sources of cfDNA/ecDNA are the dead cells [4]. Another hypothesis suggests that cfDNA/ecDNA could be actively excreted into the extracellular medium by living cells [5]. Extracellular DNA is a highly versatile physiological parameter of the body, changing under external damaging factors (e.g., radiation and toxins), during internal adverse changes in an organism (e.g., oxidative stress caused by physical activity, inflammation, and emotional stress), as well as in the different pathologies [6–12].

Сell-free DNA affects many body cells, increasing oxidative stress and activating proinflammatory cellular response [8, 13, 14]. Under oxidative stress, both nuclear and cfDNA undergo oxidative modification [7, 15–18]. The oxidized cfDNA pool increases due to the death of the cells with a high level of oxidative irreparable DNA damage. Oxidative stress induces oxidation of GC-rich sequences in the cfDNA, affecting the content of the oxidation marker 8-oxodG in the cfDNA [18]. Oxidative stress leads to the oxidation of all DNA nucleobases to varying degrees; however, the main products of DNA oxidation are thymidine glycol and 8-oxo-7,8-dihydro-2′-deoxyguanosine (8-oxodG) [16–22]. The most widely used marker of oxidatively generated DNA damage is 8-oxodG [7, 17, 18].

Oxidative stress is induced in many diseases, and normal cellular reaction to stress is determined by the general mechanism of regulation for the development of an adaptive reaction. Therefore, oxidized cfDNA under oxidative stress may possibly play a role of one of the stress-signaling factors transmitting a significant signal from dead cells to the living ones [18]. Such biological role of cfDNA signaling is associated with signaling about damage or any other adverse effects that induce oxidative stress and consequent cell death [23]. Previously, we showed that during oxidative stress oxidized cfDNA may be transferred into the cancer and stem cells [24, 25]. Transfer of oxidized cfDNA into the cells leads to the induction of the free radical production, oxidation of nuclear and mitochondrial DNA, and antioxidant protection of cells through activation of а master regulator of antioxidant system—NRF2 enhancement [14, 24–27].

DNA oxidative modification in the brain may alter stress response during stressful conditions and mental disorders. 8-oxodG level increases in plasma cfDNA in schizophrenic patients [7]. The increased level of oxidative modifications was found in the postmortem brains of schizophrenia patients [28]. Oxidative stress may lead to the DNA oxidation in brain cells. However, there is no direct evidence of this process. Because of oxidatively generated DNA damage, part of the brain cells may die, and oxidized cfDNA fragments are consequently released in the extracellular space. Little is known on how brain cells react to oxidized cfDNA.

In the present study, we investigated an effect of the oxidatively generated DNA modification on the cfDNA ability to be transferred into the cultured neurons and glial cells, oxidized cfDNA effects on 8-oxodG production, and the antioxidant NRF2 signaling pathway activation. In addition, we analyzed stress effect on the increase of 8-oxodG levels in the blood plasma cfDNA, PBL, and hippocampus of stressed rats using *in vivo* experiments.

## 2. Materials and Methods

### 2.1. DNA Isolation and Preparation of Oxidized DNA Samples In Vitro

Isolation of genomic DNA from blood and brain samples and cell-free DNA from plasma samples was carried out by a standard method of extraction with organic solvents. Genomic DNA concentration was determined spectrophotometrically; cfDNA concentration was determined fluorimetrically using PicoGreen (Invitrogen). The relative standard error of the genomic DNA concentration measurement was 3%. Oxidized DNA samples were prepared from the selected genomic DNA sample by combined treatment of 300 mM H_2_O_2_ and UV light (wavelength *λ* = 312 nm, 1.5 min, 25°C) (DNAoxy). The modified DNA was precipitated with two ethanol volumes in the presence of 2 M ammonium acetate, washed twice with 75% ethanol, then dried, and dissolved in water. According to data of the ESI-MS/MS method, the content of 8-oxodG in model fragments was 1200 8-oxodG per 10^6^ deoxynucleosides or 1200 AU, equivalent to the actual level of 8-oxodG in the cfDNA samples under severe oxidative stress. It is assumed that the genomic DNA oxidation by H_2_O_2_*in vitro* eliminates the effects of other possible factors influencing the cfDNA properties, such as changes in the methylation level or other different sequence contents.

Labeled genomic DNA fragments (DNAgreen) were obtained by nick translation using Spectrum Green fluorescent labels [27]. Labeling of extracted genomic and plasmid DNA was performed by nick translation using a CGH Nick Translation Kit (Abbott Molecular) under the manufacturer's protocol with slight modification. Solutions of genomic and plasmid DNA (3 *μ*g/*μ*L) were labeled with Spectrum Green. In the reaction mix, 50% of the dTTP was substituted with the labeled dUTP. About 20% of the fluorescent-labeled nucleotide was incorporated into the DNA, while unincorporated nucleotides were removed by ethanol precipitation. The fragment size was in a 300–3000 bp range as determined by electrophoresis in 1% agarose.

Oxidized labeled DNA (DNAoxyGreen) was synthetized by annealing of denatured labeled genomic DNA samples and oxidized *in vitro* DNA mixture.

Oxidized labeled DNA (DNAoxyGreen) was synthetized by annealing of denatured labeled genomic DNA samples and oxidized *in vitro* DNA mixture. DNAGreen (100 ng/mL) and DNAoxy (100 ng/mL) were heated to 75°С in 70% formamide-PBS and slowly cooled to 42°C using the StepOnePlus (Applied Biosystems), then kept at 37°С for a few hours.

### 2.2. Plasmid Construction

Plasmid pEGFP-C1 that contains the EGFP gene (http://www.bdbiosciences.com, GenBank accession number U55763) was used as a vector. The DNA fragment to be inserted was synthesized and consisted of 59 base pairs flanked with BamHI restriction sites (italics) and containing the poly-G (underlines) sequence. Poly-G inserts are the target for oxidation and make pEGFP-Gn oxidizing construct.

In order to obtain the DNA fragment, PCR with primers GF_601 gggcccgggatccaccggatctagataatcgccgtcccgcccgccgcctt and C10 tttttggatccccccccccccaaggcggcgggcgggacggcga were used.

The fragment was purified by agarose gel electrophoresis and treated with BamHI. The vector pEGFP-C1 was treated with BamHI and added to the DNA fragments with subsequent ligation with T4 DNA ligase. Competent E. coli (strain JM110) cells were then transformed and grown on LB with agarose and kanamycin (50 *μ*g/mL). The clones were analyzed by PCR with oligonucleotides R_SEQ_N and SEQ_C in order to confirm the insertion of the DNA fragment (SEQ_C = catggtcctgctggagttcgtg, R_SEQ_N = caataaacaagttaacaacaacaattgc). Selected clones were grown in liquid medium, and plasmids were isolated. After confirmation of the designed DNA sequence by sequencing, the plasmids were extracted using an Invisorb Plasmid Maxi Kit (Invitek GmbH, Germany). Oxidized and model cfDNA fragments were added to the cell culture in 50 ng/mL concentration for 24 h.

### 2.3. Animals

**Figure 1:**
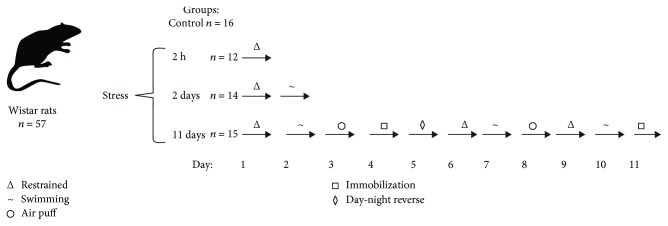
Experimental design. 57 male Wistar rats were divided into four groups: control and three stressed groups. The figure shows the types of stressors applied for each group.

57 male Wistar rats (250-300 g) were used in experiments. Animals were housed 3-4 per cage in standard laboratory conditions under a 12 h light/dark cycle at 20-22°C and ~50% humidity; food and water were provided ad libitum. A study was performed in accordance with the European Communities Council Directive of 24 November 1986 (86/609/EEC). All rats were divided into four groups (Figure 1): (1) control (*n* = 16); (2) rats stressed for 2 h (*n* = 12, group “2 h stress”); (3) stressed for 2 days (*n* = 14, group “2 days stress”), and (4) animals subjected to chronic unpredictable stress for 11 days (*n* = 15, group “11 days stress”). The rats from the second group were restrained in a plastic restrainer cage (180 × 70 × 70 mm) for 2 h. The rats from the third group were stressed for 2 days: in the first day, they were restrained in the cage for 1 h, and in the 2nd day, they were subjected to forced swimming-induced stress in water (~20°C). The rats from the fourth group were stressed for 11 consecutive days. Each day, all of them were subjected to one of the following procedures as part of chronic unpredictable stress protocol: (a) restrained for 1 h in the restrainer cage, (b) immobilized by stretching on board and putting tape on limbs for 1 h, (c) air puff stress for 30 min, (d) forced swimming-induced stress for 20 min in water (~20°C), and (e) subjected to abrupt change of day-night-light pattern for 24 h. All types of stressors were described previously in our own experiments and in the literature [29, 30]. Those stressors changed every day during 11 days of an experiment to avoid development of an adaptation to similar chronic exposure. 1 h after the last stress, all animals were sacrificed, and at the time of sacrifice, the animals were lightly anesthetized with ether. Blood samples were centrifuged in the containers with heparin.

### 2.4. Brain Section Preparation

After decapitation, the brains were removed and rapidly frozen in cooled isopentane at –45°C. Coronal brain sections with 10 *μ*m thickness were cut on a cryostat. The sections were thawed onto glass slides, fixed for 10 min in 4% paraformaldehyde solution (Sigma, St. Louis, USA), and rinsed 3 times in phosphate-buffered saline.

### 2.5. Сell Fixation

Immunofluorescence analysis of cerebellum cells was performed by appropriate antibodies after fixation. Cells were fixed in 2% PFA (4°C, 20 min), washed with PBS, and then permeabilized with 0.1% Triton X-100 in PBS (15 min, 4°C), followed by blocking with 0.5% BSA in PBS (1 h, 4°C).

### 2.6. Fluorescence Microscopy

Culture cells and brain sections were incubated overnight with mouse monoclonal antibody against 8-oxodG (sc-66036, Santa Cruz) and NRF2 rabbit polyclonal antibody bs-1074R-FITC (BIOSS, USA). After washing with PBS with 1% BSA, they were incubated for 2 h (20°C) with m-IgGk BP-PE phycoerythrin conjugated (sc-516141, Santa Cruz) and then stained with DAPI. The antibody concentration was applied in accordance with the manufacturer's recommendations. Images were obtained using an AxioScope A1 microscope (Carl Zeiss) or confocal microscope (Leica TCS SP8).

### 2.7. Flow Cytometry

Peripheral blood lymphocytes (PBL) were isolated from 15 mL of blood. PBL were separated by centrifuging at 1500 rpm for 30 min using ficoll (density 1.09 g/mL, Paneco) for lymphocyte separation and following the methods of a previously published study [7].

### 2.8. 8-oxodG Quantitation

The content of 8-oxodG in сfDNA was determined using immunoassay. The membrane (Extra C) was moistened with 20х SSC solution and dried. CfDNA (10 ng/mL) solution in TE-buffer was applied to the prepared filter in an amount of 2 mL per dot. Three to five dots were applied from each sample. To the same filter, we applied standard samples of oxidized genomic DNA with a known 8-oxodG content in order to plot a calibration curve of a signal intensity dependence on the 8-oxodG content in the sample. Standard samples of human oxidized genomic DNA were obtained via reaction of DNA with hydrogen peroxide as previously described [27]. The content of 8-oxodG in the control samples was determined by the ESI-MS/MS method using the AB SCIEX 3200 Qtrap machine. The filter was heated at 80°С in a vacuum for 30 min. The membrane was blocked (30 min, 30°С) in a solution (0.1% fat-free milk, 0.1% gelatin, tris-HCL buffer, pH 7.5, 0.1 M sodium chloride). Then, the membrane filter was treated for 30 min (25°C) with a conjugate of biotin with an antibody (sc-66036, Santa Cruz) to 8-oxodG (1 mg/mL) in solution A (0.1 M tris-HCL buffer, pH 7.5, 0.1 M sodium chloride). The filter was washed (3 × 10 min) with solution A. Then, the membrane filter was treated for 20 min (25°С) with a conjugate of streptavidin with alkaline phosphatase (1 mg/mL, Sigma) in solution B (0.1 M tris-HCL buffer, pH 7.5, 0.1 M sodium chloride, 0.005 M magnesium chloride). The filter was washed (3 × 10 min) with solution B. Then, the filter was placed to a solution of substrates for alkaline phosphatase (tris-HCL buffer, pH 9.5, 0.1 M sodium chloride, 0.005 M magnesium chloride, 4.4 mL/mL NBT, and 3.3 mL/mL BCIP). Reaction was conducted in the dark at 37°С under visual control of stained violet dot emergence. After the reaction had been finished, the filter was washed with water and dried in the dark. The dried filter was scanned. For the quantitative analysis of the dots, a special software was used (Images6, Research Centre for Medical Genetics, Moscow). The software determines the dot location, measures the nearest background signal, and calculates the integral dot intensity. Signals from several dots for the same sample were averaged. The mean and standard error were calculated. The 8-oxodG content in a studied sample is determined using the calibration curve equation. The relative standard error of the index 8-oxodG was 15 ± 5%.

### 2.9. Culture of Rat Cerebellum Cells

**Figure 2:**
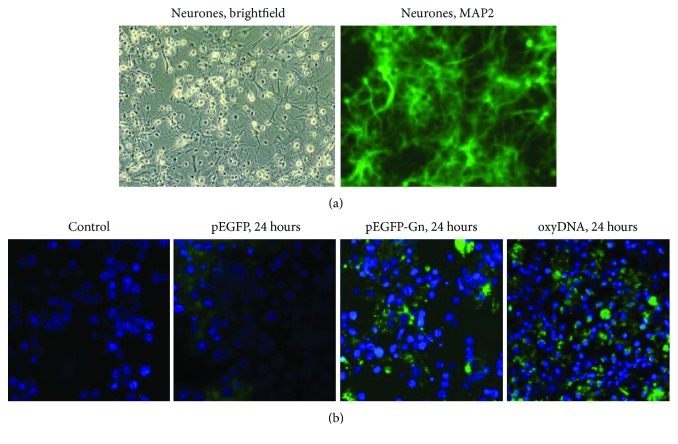
(a) MAP-2 detection in primary cell culture of Wistar rat cerebellum. MAP-2 (FITC) in neurons. Nuclei are stained with DAPI. ×40. (b) Staining of primary cell culture of Wistar rat cerebellum cells with the various types of the labeled DNA fragments. From left to right: control, pEGFP, pEGFP-Gn, and DNAoxyGreen. Nuclei are stained with DAPI. ×40.

Granular neurons of the cerebellum obtained from 8-9-day-old Wistar rats with the glial cell preservation were used in experiments. The use of the animals was approved by the Ethics Committee of the Institute. Preparation, sowing, and manipulation on the cell cultures were carried out in sterile conditions. After decapitation of rats, the cerebellums were separated surgically and transferred in a Hanks salt solution (Paneco, RF), then were washed with DMEM+Hepes (Paneco, RF); solution Versene+EDTA+Trypsin 0.25% (Paneco, RF) was used for lysis in a thermostat at 37°C for 15 min. Dulbecco's solution (DPBS; Paneco, RF) was added to stop the lysis. 1 mL Pasteur Pipette (Paneco, RF) was used for suspension. The suspension contained about 2 million cells per 1 mL. Sowing was carried out on 6- and 24-well plates covered with poly-D-lysine (applied in 50 *μ*g/mL solution for 1 h, then washed twice with deionized water, and dried in a laminar flow hood). Adapted medium for the cultivation of neurons (Neuromed) (the main components: Neurobasal medium (NBM)+“Neuromax” (Paneco, RF)) was used as a nutrient medium. The cell culture was kept for 72 h in the incubator (CO_2_ 5%, air 95%, 37°C, Hum = 95%). Complete replacement of the nutrient medium was performed, as well as a visual assessment of neuronal recovery in cell culture (AxioVert microscope, Carl Zeiss Microscopy, Germany) before each test. The analysis of cell morphology was carried out with light microscopy (Figure 2(a) (Neurones, brightfield)). Cell cultures were analyzed after the addition of neuronal-specific antibodies (MAP-2, SC 135979, Santa Cruz) (1 h, 25°C) followed by secondary fluorescence-labeled antibodies (Alexa Fluor 488) with the AxioVert fluorescence microscope (Carl Zeiss Microscopy, Germany) (Figure 2(a) (Neurones, MAP2)). Experiments on the cell cultures were repeated at least 5 times for each neuronal isolation. Isolation of neurons from biological material was also repeated at least 5 times.

### 2.10. Quantification of mRNA Levels

Total mRNA was isolated from rat cerebellum cell cultures and PBL using RNeasy Mini kits (Qiagen, Germany), treated with DNAse I, and reverse transcribed by a Reverse Transcriptase kit (Sileks, Russia). The expression profiles were obtained using qRT-PCR with SYBR Green PCR Master Mix (Applied Biosystems). The mRNA levels were analyzed using the StepOnePlus (Applied Biosystems); the technical error was approximately 2%. The following primers were used (Sintol, Russia): *Nrf2* (F: CTACTCCCAGGTTGCCCACA; R: CGACTCATGGTCATCTACAAATGG), *Hmox1* (F: TCGACAACCCCACCAAGTTC; R: AGTATCTTGAACCAGGCTAGCA), and *Tbp* (reference gene) (F: TGCACAGGAGCCAAGAGTGAA; R: TTCACATCACAGCTCCCCAC).

The standard curve method was used for the quantification of RNA levels.

### 2.11. Statistics

The statistical data analysis was conducted using MS Excel, Statistica 10.0, StatGraphics software. The null hypotheses of the absence of the difference between the compared samples were tested with the Mann–Whitney *U* test. The dynamics of the parameters were analyzed using the Kruskal-Wallis ANOVA tests. Samples were considered significantly different at *p* < 0.05.

## 3. Results

### 3.1. Oxidatively Damaged cfDNA May Be Transferred into the Cultured Neurons

Previously, we have shown that the ability of oxidatively damaged cfDNA to be transferred into human cancer and stem cells significantly depends on their oxidative modifications [14, 24, 25, 27] and identified signaling pathways, activated by oxidized cfDNA in different human cells [14, 18, 25, 31]. We suggested that oxidized cfDNA, produced by dying cells under endogenous or exogenous oxidative stress, is able to be transferred into neurons and glial cells, activating signaling pathways genes. Since it is known that under oxidative stress the permeability of the blood-brain barrier increases [32], cfDNA may be transferred into the brain cells and accordingly appear in the intracellular space due to the death of the part of the glial cells participating in a protective barrier [33]. The presence of fetal DNA in the cerebrospinal fluid of pregnant women indicates the possibility of DNA to cross the blood-cerebrospinal fluid barrier [34]. According to our hypothesis, oxidized cfDNA may be transferred into neurons and glial cells and affect their genetic apparatus, causing oxidatively generated DNA modifications in the nuclei and activating the transcription of genes involved in antioxidant response.

It is shown that oxidative cfDNA modification promotes its penetration into the neurons and glial cells (Figure 2(b)). In 24 h, labeled oxidized cfDNA fragments (DNAoxyGreen) were transferred into the cytoplasm of 80% of the cells. The nonoxidized fragments most probably were not transferred into the cytoplasm, since after the addition of unoxidized labeled DNA fluorescence was observed only in nonfixed cells, disappearing after fixation and washing. Incubation of neurons and glial cells with pEGFP-Gn and pEGFP vectors, containing the fluorescent GFP protein gene, within 24 h led to an increase in the fluorescence in the cells incubated with plasmid compared with intact control. At the same time, an addition of genetic structures containing oxidized inserts increased the fluorescence in cells by 30-40% (*p* < 0.01): pEGFP-Gn plasmid was transferred into the cytoplasm of 70-75% of cells and vector into the cytoplasm of 30-45% of cells (see Figure 2(b)). Therefore, the fluorescence increases in the following order: control ≤ DNAGreen < pEGFP<<pEGFP − Gn≤≤DNAoxyGreen. Therefore, the higher the oxidation is, the easier oxidized cfDNA fragments are transferred into the cultured brain cells.

**Figure 3:**
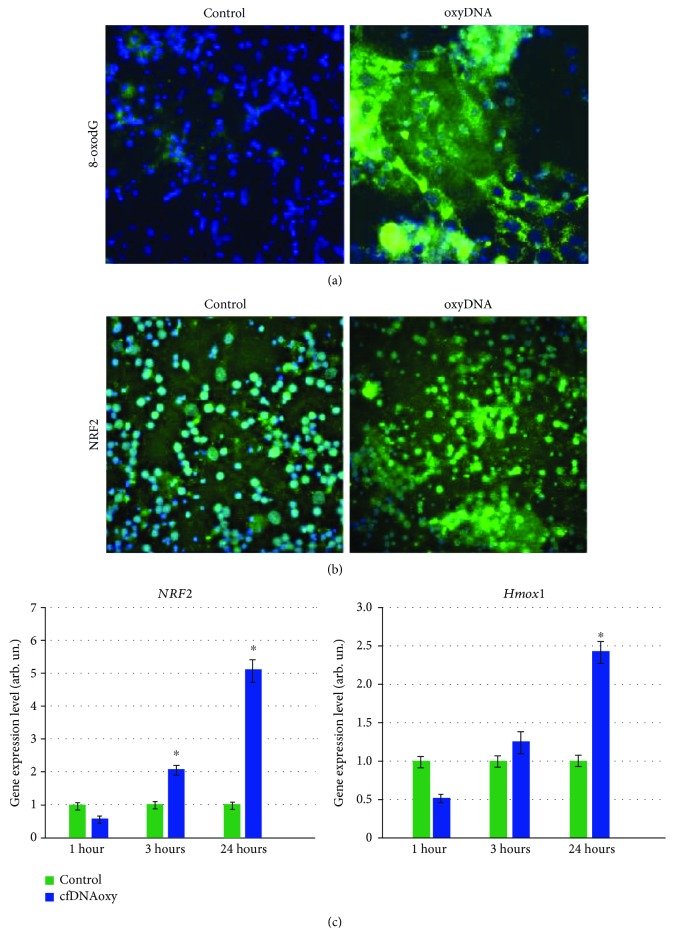
(a) Staining of primary cell culture of Wistar rat cerebellum with 8-oxodG-antibody (PE). From left to right: control and oxyDNA-treated cells (180 min). Nuclei are stained with DAPI. ×40. (b) Staining of primary cell culture of Wistar rat cerebellum with NRF2-antibody (FITC). From left to right: control and oxyDNA-treated cells (180 min). Nuclei are stained with DAPI. ×40. (с) Oxidized cfDNA (50 ng/mL) effect on *Nrf2* and *Hmox1* expression in the mixed culture of neurons and glia. The incubation time is shown on the graph. Expression was detected by RT-PCR relatively to housekeeping gene TBP. ^∗^*p* < 0.001 vs. the control group.

Recently, we have shown that oxidized cfDNA penetration into the cells is accompanied by the induction of the reactive oxygen species (ROS) synthesis [14, 24, 25, 27] that may lead to the oxidatively generated DNA modifications in cultured cells. An addition of oxidized cfDNA fragments (concentration 50 ng/mL, cultivation time 2 h) to the culture medium increases the 8-oxodG level in cultured cells by 5-6 times (*p* < 0.001; Figure 3(a)).

Therefore, oxidized cfDNA, penetrating into neurons and glial cells, induces an increase in the level of cell nucleus oxidative damage. Such increase of oxidative damage induced by oxidized cfDNA at a concentration of 50 ng/mL results in the activation of the antioxidant response master regulator—transcription factor NRF2 transcription and translation in neurons and glial cells. 1 h later, the level of *Nrf2* gene expression increases 2 times (*p* < 0.001) and 24 h later 5-6 times (*p* < 0.001, Figure 3(b)). 24 h later, the expression of *Hmox1* gene encoding hemoxygenase 1 increases 2-2.5 times (*p* < 0.001). *Hmox1* gene transcription correlates with NRF2-signal pathway activation (Figure 3(c)).

The increase in the *Nrf2* expression is followed by an increase of the corresponding protein synthesis. 3 h after oxidized cfDNA addition (concentration 20-50 ng/mL), we found an accumulation of the NRF2 protein in neurons and glial cells followed by its gradual transport into the cell nucleus (Figure 3).

Thus, we have shown that cfDNA is able to penetrate into the mixed culture neurons and glial cells. The higher the oxidation level is, the more active is its transport into the cells. Oxidized cfDNA fragments induce glial cells and neurons oxidatively generated DNA damage. It activates the antioxidant reactions, leading to the induction of transcription factor NRF2, followed by an activation of NRF2 signaling pathway genes—rise of *Nrf2* and *Hmox1* gene expression and NRF2 protein synthesis.

Therefore, under oxidative stress, the сfDNA with increased oxidative modifications may be transferred into the neurons and glial cells, activating protective antioxidant mechanisms. In our opinion, the current literature is almost lacking of evidence that oxidized cfDNA fragments may affect cell viability and activate the antioxidant system in neurons. There was a study where increased oxidized DNA-based (8-oxodG) level in urine or in the brain (hippocampus and frontal cortex) of stressed animals was not detected, while there was a tendency to increase expression of genes involved in DNA repair, possibly reflecting an activation of compensatory mechanisms in response to ROS damage [35]. Another report showed an increase in expression of oxidative stress markers (8-oxodG and nitrotyrosine) and *Nox2* mRNA in the hypothalamus of rats after two weeks of chronic psychosocial stress [36].

In continuation of our study on the cultured neurons and glial cells, we analyzed cfDNA characteristics, particularly concentration and 8-oxodG level, in experimental stress models *in vivo*. In the present study, we applied acute, subchronic, and unpredictable chronic stress in rats that were able to activate different cellular signaling pathways leading to an increase in the ROS production [37, 38].

### 3.2. Blood Plasma cfDNA and cfDNA 8-oxodG Concentrations in Rats under Stress

**Figure 4:**
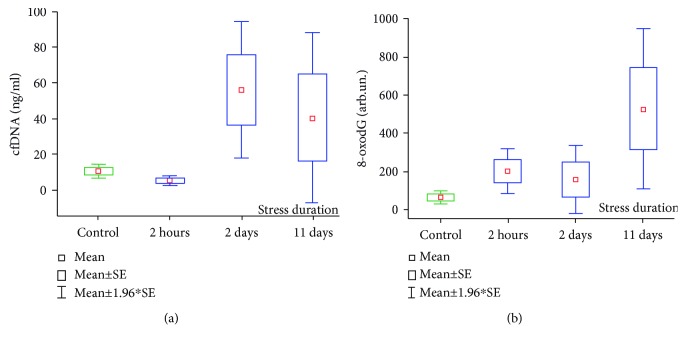
(a) Cell-free DNA concentrations in plasma samples of rats subjected to acute and chronic unpredictable stress. Kruskal-Wallis test: *H* (3, *N* = 38) = 9,297; *p* = 0.0256. (b) 8-oxodG content in plasma cfDNA samples of rats subjected to acute and chronic unpredictable stress. Kruskal-Wallis test: *H* (3, *N* = 38) = 12,231; *p* = 0.0066. The data are presented in arb. un.; 1 arb.un = 1 molecule 8-oxodG per 10^6^ deoxynucleosides.

As it is shown on Figure 4(a), plasma cfDNA concentration increased significantly during the stress exposure. The mean cfDNA concentration in control rats was 10.4 ± 2.0 ng/mL vs. 56.1 ± 19.6 ng/mL in the group “2 days stress” (*p* = 0.095; Mann–Whitney *U* test) and 40.4 ± 24.3 ng/mL in the group “11 days stress” (*p* = 0.049; Mann–Whitney *U* test). It has been noticed that rats subjected to the acute immobilization stress (group “2 h stress”) demonstrated a twofold decrease in their plasma cfDNA concentrations (5.1 ± 1.4 ng/mL) compared to controls, but it was not significant (*p* = 0.1105). There was a similar change in plasma cfDNA concentrations we observed in healthy volunteers who participated in 17-day isolation study subjected to immobilization (project “SIRIUS 17/19”) [39]. Statistical analyses using the Kruskal-Wallis ANOVA test revealed a significant increase in plasma cfDNA corresponding with stress duration (see Figure 4(a)).

8-oxodG content in plasma cfDNA samples of rats subjected to acute and chronic stress is shown in Figure 4(b). It was found that both acute and chronic stress exposures were associated with the 2.5-8.0-fold increase of 8-oxodG content in cfDNA samples. Therefore, the mean 8-oxodG level in the control animals was 64.7 ± 15.4 AU vs. 201.6 ± 60.2 AU in the group “2 h stress” (*p* = 0.0233; Mann–Whitney *U* test), 159.9 ± 90.2 AU in the group “2 days stress” (*p* = 0.2439; Mann–Whitney *U* test), and 526.0 ± 215.4 AU in the group “11 days stress” (*p* = 0.0037; Mann–Whitney *U* test). Statistical analyses using the Kruskal-Wallis ANOVA test revealed a significant increase of 8-oxodG content in plasma cfDNA samples corresponding with stress duration (see Figure 4(b)).

### 3.3. 8-oxodG and NRF2 Protein Expression in Peripheral Blood Lymphocytes

**Figure 5:**
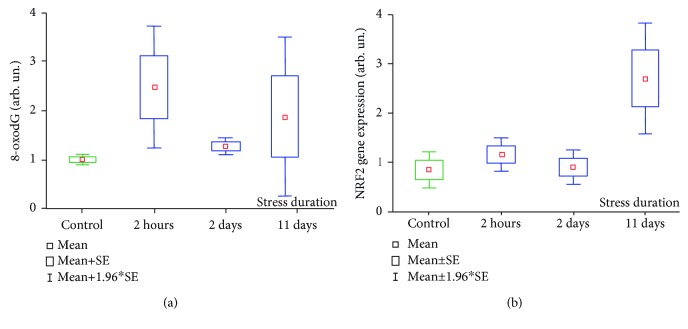
(a) 8-oxodG in PBL of rats subjected to stressor exposures. Kruskal-Wallis test: *H* (3, *N* = 38) = 5,219; *p* = 0.1565. (b) NRF2 protein expression in PBL of rats subjected to stressor exposure. Kruskal-Wallis test: *H* (3, *N* = 38) = 10,193; *p* = 0.0170.

Flow cytometry was applied to measure 8-oxodG content and NRF2 protein expression in peripheral blood lymphocytes (PBL) obtained from rats subjected to the different stress exposures. The data is shown on the Figure 5. As it can be seen in Figure 5(a), the dynamics of 8-oxodG content in PBL of stressed rats differed from those of 8-oxodG in plasma cfDNA samples. Statistical analyses using the Kruskal-Wallis ANOVA test did not reveal significant changes of PBL 8-oxodG level in rats after different stress durations. For the group of rats subjected to 2 h restraining, the highest value of the mean 8-oxodG content in PBL has been found (2.47 ± 0.6 AU vs. 1.00 ± 0.05 AU in the control group). However, the differences between this group and controls were not significant due to the high value variability (*p* = 0.1437; Mann–Whitney *U* test). The mean levels of PBL 8-oxodG in rats stressed for 2 and 11 days were 1.26 ± 0.09 AU (*p* = 0.0338) and 1.40 ± 0.29 AU (*p* = 0.1643; Mann–Whitney *U* test), respectively.

NRF2 protein expression under stress according to the Kruskal-Wallis ANOVA test increased significantly (Figure 5(b)). The mean NRF2 protein content in control rat PBL was 0.84 ± 0.19 AU vs. 1.15 ± 0.17 AU in the group “2 h stress” (*p* = 0.210), 0.90 ± 0.18 AU in the group “2 days stress” (*p* = 0.942; Mann–Whitney *U* test), and 2.69 ± 0.57 AU in the group “11 days stress” (*p* = 0.016; Mann–Whitney *U* test).

### 3.4. 8-oxodG in the Hippocampus

**Figure 6:**
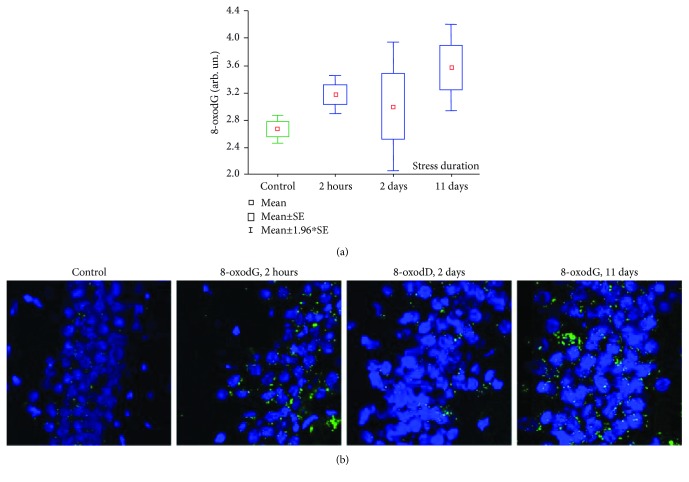
(a) 8-oxodG in hippocampal samples of rats subjected to stress. Kruskal-Wallis test: *H* (3, *N* = 76) = 9.334; *p* = 0.0252. (b) Staining of rat hippocampal dentate gyrus stained with 8-oxodG antibodies (green). Nuclei are stained with DAPI (blue). ×40.

According to the immunoassay analysis, 8-oxodG content in hippocampal homogenates of rats subjected to 2 h and 11 days stress exposures increased significantly compared to control animals (Figure 6(a)). The mean 8-oxodG content in control rat hippocampus was 2.67 ± 0.11 AU vs. 3.17 ± 0.14 AU in the group “2 h stress” (*p* = 0.019) and 3.57 ± 0.32 AU in the group “11 days stress” (*p* = 0.011; Mann–Whitney *U* test). The 8-oxodG content in the hippocampus of rats stressed for 2 days was higher than that in the control group (3.0 ± 0.48 AU), but the difference was not statistically significant.

Similarly, we found an increased number of 8-oxodG-positive cells in the hippocampal dentate gyrus of rats subjected to 2 h and 11 days of stress (Figure 6(b)).

## 4. Discussion

The metabolic processes in the cells of living organisms result in a sustained ROS generation that are involved in important physiological functions at low concentrations, including signaling and regulatory mechanisms [40, 41]. Under negative environmental factors and, as a result, of endogenous oxidative stress, ROS may be generated in quantities exceeding the protective capacity of the antioxidant systems, leading to biomacromolecule damage [42]. DNA bases are also subjected to the oxidative modification with the production of 8-oxo-7,8-dihydro-2′-deoxyguanosine (8-oxodG), the most common and studied product of DNA oxidation [43, 44]. In addition, 8-oxodGTP is formed in the nucleotide pool by both 8-oxodG metabolism and endogenous reactive oxygen species [45]. Deoxyguanosine in the DNA composition has the lowest redox potential and is easily oxidized in the C8 position, leading to the 8-oxodG production followed by oxidation of 8-oxodG [46, 47]. The cytoplasmic dGTP pool is oxidized more easily than guanine in the DNA of the cell nuclei [41]. 8-oxodG is one of the main biomarkers of oxidative stress. The result of oxidatively generated DNA damage is the formation of the chromatin breaks, which may lead to the cell death when the DNA repair system is imperfect. Oxidized extracellular DNA fragments occur in the extracellular space because of the cellular death. Increased oxidized cfDNA level may be observed as a result of the different diseases and under stress. In the present study, we found a significant increase of the oxidative cfDNA modifications in Wistar rats subjected to 2 h and 11 days of stress.

The hydrolases convert 8-oxodGTP to 8-oxodGDP; 8-oxodGDP is dephosphorylated to 8-oxodGMP and then to 8-oxodGuo by a nucleotidase or phosphatase [48]. The cell membranes are permeable for 8-oxodGua, but whether the cell membrane is permeable for 8-oxodG in the cfDNA composition is still unclear [49]. We have shown previously that oxidized cfDNA may enter into cancer cells through their membranes [27]. In the present study, we investigated the fundamental oxidized cfDNA possibility to penetrate the membranes of the brain cells. We have shown that the oxidized cfDNA is able to be transferred in the cultured cells—neurons and glial cells from rats cerebellum, increasing the level of 8-oxodG in these cells. It may be the result of ROS synthesis induction in that cells, or possibly, the oxidized DNA accumulates in them. First, it is believed that the oxidative deoxyguanosine modifications in DNA are exclusively genotoxic and mutagenic and, therefore, harm nucleic acids [50, 51]; second, it is assumed that easily oxidized G in the cfDNA composition may act as the intracellular antioxidant [41].

A large number of studies are devoted to the G oxidation in the genomic and mitochondrial DNA under oxidative stress, but there is increasing interest in the study of the biological effects of exogenous 8-oxo-2′-deoxyguanosine [52]. It is known that exogenous 8-oxodGua *in vitro* protects thymidine both in free form and in the oligodeoxynucleotide composition from the oxidative degradation [52].

*In vitro* experiments had shown that 8-oxo-7,8-dihydro-2′-deoxyguanosine (8-oxodG) antioxidant activity is higher than ascorbate, uric acid, N-acetyl-L-cysteine, and superoxide dismutase [53]. Antiallergic and immunosuppressive 8-oxodG effects have been found in mice sensitized with ovalbumin [54, 55]. In an acute experimental autoimmune encephalomyelitis (EAE) in mice, 8-oxodG alleviated disease severity [56]. Thus, based on the numerous evidences that prove the positive 8-oxodG role, it was suggested that oxidized extracellular DNA fragments, penetrating into brain cells, may activate antioxidant signaling pathways in cells.

One of the key master regulators of antioxidant response is NRF2. The nuclear factor erythroid 2-related factor 2 (NRF2) is a relevant basic leucine zipper transcription factor that is essential for the regulation of cell cycle homeostasis, cytoprotection, and innate immunity of cells under stressful conditions [57, 58]. NRF2 activation leads to increased target gene expression of proteins involved in the antioxidant cellular response. We have shown that oxidized cfDNA fragments activate transcription of the master regulator of antioxidant response *Nrf2* at the gene and protein level and of the *Hmox1* gene—the target of the NRF2 signaling pathway in the cultured neurons and glial cells.

Since oxidized cfDNA fragments are able to penetrate into the cells, it was suggested that an accumulation of the oxidative cfDNA modifications under stress might affect the cells of the body—e.g., peripheral blood lymphocytes and brain cells. It was shown that the amount of oxidative modifications increases in the peripheral blood lymphocytes depending on the severity of acute and chronic stress in the experimental model.

Evidently, mammalian organisms are tolerant to the high levels of 8-oxodG in the nuclear and mitochondrial DNA [59]. It was shown that even exceeding the 8-oxodG level by 7-20 times did not lead to mitochondrial dysfunction or an increase in the frequency of tumor formation in tissues [59], probably due to the antioxidant system activation in the body. The transcription factor NRF2 in normal cells is present in the inactivated state, in combination with the cytoskeleton protein KEAP1, and is located predominantly in the cytoplasm [60–64]. When KEAP protein is modified by the reactive oxygen species, NRF2 is released from association with KEAP and moves to the nucleus, where it interacts with the antioxidant response elements of genes encoding the detoxification enzymes and cytoprotective proteins, including NADPH:quinone oxidoreductase-1 (NQO1) and hemoxygenase-1 (HMOX1) [60–64]. Acute stress, probably, releases NRF2 that is present in the inactive state; meanwhile, chronic stress depletes the content of the bound NRF2 form and increases the *Nrf2* expression.

We also analyzed in our *in vivo* experiments how the acute and chronic stress correlating with an increased 8-oxodG content in the cfDNA affects the 8-oxodG accumulation in the hippocampal cells, since the hippocampus is one of the most vulnerable brain structures involved in stress [65]. It was shown that the 8-oxodG level in the hippocampal cells increases 2 h after acute stress and then decreases, possibly after the release of protein-regulated NRF2 and activation of the antioxidant system. However, under the chronic stress, the endogenous antioxidant system becomes unable to maintain the balance of the pro/antioxidant systems, leading to the development of oxidative stress in the brain and, as a result, to an increase of the 8-oxodG level in the hippocampal dentate gyrus. The shift of that equilibrium in the biological oxidation system towards oxidative damage of brain tissues is one of the key factors in the pathogenesis of the nerve tissue damage in a variety of pathological processes [66, 67].

Therefore, we have shown that the oxidized extracellular DNA may be a marker not only of the pathological process but also of the body adaptive stress response. Participating in the different complex physiological and biochemical functions, such as induction of cellular DNA oxidative modifications and activation of antioxidant defense mechanisms, the oxidized cfDNA may play both a destructive role, leading to the DNA breaks in the cells and, therefore, to the chromatin rearrangements, and a positive role, activating the antioxidant system. ROS appearance may cause DNA damage and activate the antioxidant system in the cell population *in vitro*, leading to the development of an adaptive response in the cells and increasing their tolerance to subsequent damaging effects. We have shown that previously for cancer cells [27] and mesenchymal stem cells [24]. The further investigation of the oxidized cfDNA ability to pass the blood-brain barrier and activate the complex cascades of signaling pathways in the brain cells will lead to the understanding of the mechanisms of the body's response to stress and the identification of new stress moderators.

## 5. Conclusion

**Figure 7:**
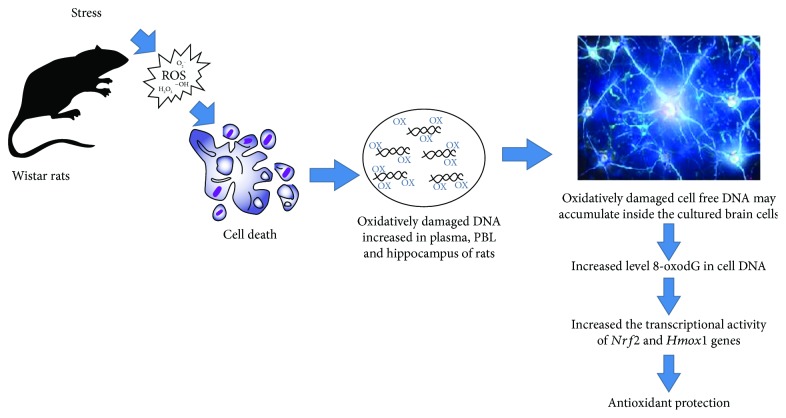
Schematic representation of the extracellular DNA participation in the antioxidant response development. Explanations in the text.

The role of oxidized cfDNA in the stress and pathological processes is still unclear. In the present study, we have shown that oxidized DNA fragments may accumulate inside the cultured brain cells and alter the transcriptional activity of *Nrf2* and *Hmox-1* genes, the important factors of antioxidant protection (Figure 7). In parallel *in vivo* experiments, we have demonstrated that the levels of oxidized DNA modifications significantly increased in the plasma, PBL, and hippocampus of rats subjected to unpredictable chronic stress (Figure 7). The supposed possibility of the oxidized DNA fragment exchange between different tissues and organs including the brain in our opinion should be studied in the future. Therefore, our study may be a starting point in the effort to examine the cfDNA and the oxidized DNA modification role in the homeostatic and pathological processes.

## Data Availability

The data used in this study are available from the corresponding author upon request.
